# Smooth 2D manifold extraction from 3D image stack

**DOI:** 10.1038/ncomms15554

**Published:** 2017-05-31

**Authors:** Asm Shihavuddin, Sreetama Basu, Elton Rexhepaj, Felipe Delestro, Nikita Menezes, Séverine M Sigoillot, Elaine Del Nery, Fekrije Selimi, Nathalie Spassky, Auguste Genovesio

**Affiliations:** 1 Institut de Biologie de l’Ecole Normale Superieure (IBENS), CNRS UMR8197, Inserm U1024, Ecole Normale Superieure, PSL Research University, 46 Rue d’Ulm, 75005 Paris, France; 2 Center for Interdisciplinary Research in Biology (CIRB), College de France, CNRS UMR 7241, INSERM U1050, PSL Research University, 11 Place Marcelin Berthelot, 75005 Paris, France; 3 Biophenics, Departement de Recherche Translationnelle, Institut Curie, PSL Research University, 26 Rue d’Ulm, 75005 Paris, France

## Abstract

Three-dimensional fluorescence microscopy followed by image processing is routinely used to study biological objects at various scales such as cells and tissue. However, maximum intensity projection, the most broadly used rendering tool, extracts a discontinuous layer of voxels, obliviously creating important artifacts and possibly misleading interpretation. Here we propose smooth manifold extraction, an algorithm that produces a continuous focused 2D extraction from a 3D volume, hence preserving local spatial relationships. We demonstrate the usefulness of our approach by applying it to various biological applications using confocal and wide-field microscopy 3D image stacks. We provide a parameter-free ImageJ/Fiji plugin that allows 2D visualization and interpretation of 3D image stacks with maximum accuracy.

## Abstract (web summary)

Maximum Intensity Projection is a common tool to represent 3D biological imaging data in a 2D space, but it creates artefacts. Here the authors develop Smooth Manifold Extraction, an ImageJ/Fiji plugin, to preserve local spatial relationships when extracting the content of a 3D volume to a 2D space.

Cell biologists routinely use fluorescence microscopy to observe spatial organization of organelles, co-localization of vesicles or to study the impact of gene expression or small compounds on cell morphology^1^,^2^,^3^,^4^. Furthermore, it is common nowadays to image the same preparation repetitively at various depths to obtain a three-dimensional (3D) image under the form of a stack of two-dimensional (2D) images. Unlike a single 2D image, which can be rendered and interpreted straightforwardly on a screen, a 3D stack needs to be processed to be displayed.

An image stack can be employed to image a full 3D object. In this case, a single projection cannot be satisfactory and an interactive rendering tool must be used to obtain successive projections of the data in arbitrary directions using transparency filters. Optionally, a detection step can be introduced before the projection to focus on the reconstruction of layers of interest^5^. A variety of software programme as Voxx^6^, NIH’s Fiji/ImageJ^7^ or VTK^8^ propose such options with an interactive visualization.

Image stacks are also useful to image flattish or so-called 2.5D objects such as an epithelium, a monolayered cell culture, a membrane within an *in vivo* tissue sample or a flat biological structure such as cultured neurons. This is because at high resolution, those objects cannot hold within the depth of field of a microscope and therefore a single 2D image acquisition often suffers from being only partially in focus. In contrast, a 3D stack contains all pieces of information needed to reconstruct a focused 2D image. In this case, a projection in the *z* direction is often preferred. First, because it contains the highest amount of information. Second, because resolution is always lower in *z* than in *x*/*y* in fluorescence microscopes. Finally, because it produces a single 2D image with no need of an interactive interface.

In practice, more than 80% of the biology community who acquire 3D volumes use maximum intensity projection (MIP), one of the simplest Z projection methods, to reduce a 3D stack into a single 2D image as reflected in a survey (see ‘Methods’ section). MIP consists in retrieving the level of maximum intensity along the *z* axis for each *x*,*y* position. The image of levels is called the index map while the image made of intensity values corresponding to that index map is called the composite image or the projection. There were good reasons for MIP to be widely adopted: it is parameter free, fast and straightforward to use as it is implemented in NIH Fiji/ImageJ^7^ and in many other programmes.

Despite its widespread adoption, MIP presents a major drawback: it produces a highly discontinuous index map. It means that two consecutive pixels in the resulting image can belong, respectively, to the first and the last images of the stack, thus far apart in the original data set. Therefore, MIP produces an artificial 2D image that does not match any existing structure in the original volume. The effect is especially strong on the background or in the vicinity of the foreground producing important loss of contrast. Consequently, while MIP can be useful to obtain a global overview of the content of a stack, any image made daily this way by numerous end users are at best imprecise or not contrasted and at worst can lead to wrong interpretation or biased quantification.

While not used in practice, other Z projection methods, mostly inspired by digital photography, were proposed as alternatives to MIP. However, they also suffer from drawbacks as shown in our evaluations (see ‘Methods’ section). First, while the discontinuity issue was identified by some authors, the proposed solutions mainly consisted in smoothing the index map without distinction between the foreground and the background. The last tends to degrade the foreground and does not properly solve the issue for the background. Second, colour channels are loosely taken into account in practice, and channels are systematically processed independently. In consequence, a colour pixel in the resulting image can be made of colour components from different voxels far apart in different channels possibly leading to false interpretation in the co-localization of differently labelled objects. Third, in opposition to MIP, most of the other methods require parameters which imply making a subjective choice. Finally, whatever the methodological drawbacks, there seems to also be an accessibility issue as, to our knowledge, the only tool made easily available to end users is the extended depth of field (EDF) method by Aguet *et al*.^9^,^10^ implemented as a plugin in the popular software programme NIH Fiji/ImageJ^7^. However, this tool was specifically designed for brightfield microscopy images and still suffers from the aforementioned issues.

Here we propose a method, smooth manifold extraction (SME), that focuses on what is currently missing in the available toolkits: maintaining spatial consistency in the projection both within one channel and also between channels. Obtaining a spatial consistency between channels is straightforward and just a matter of implementation: it consists in choosing a single reference channel for extracting the index map to apply to all channels. Therefore, our approach mostly focuses on enforcing the spatial consistency of an index map obtained from a single channel. The intuitive idea is to fit a ‘smooth’, parameter-free, 2D manifold onto the foreground signal while ‘ignoring’ the background, thus propagating the index map found in the foreground to the local background. To this aim, the fitting is constrained at each pixel by minimizing together the distance from the index map to the most focused *z* level (ensuring its foreground proximity) and the local variance of the index map (ensuring its smoothness). We compare results obtained with our method and others on synthetic and real data sets covering a variety of biological objects morphology for a variety of applications.

## Results

### SME improves spatial consistency

**Figure 1:**
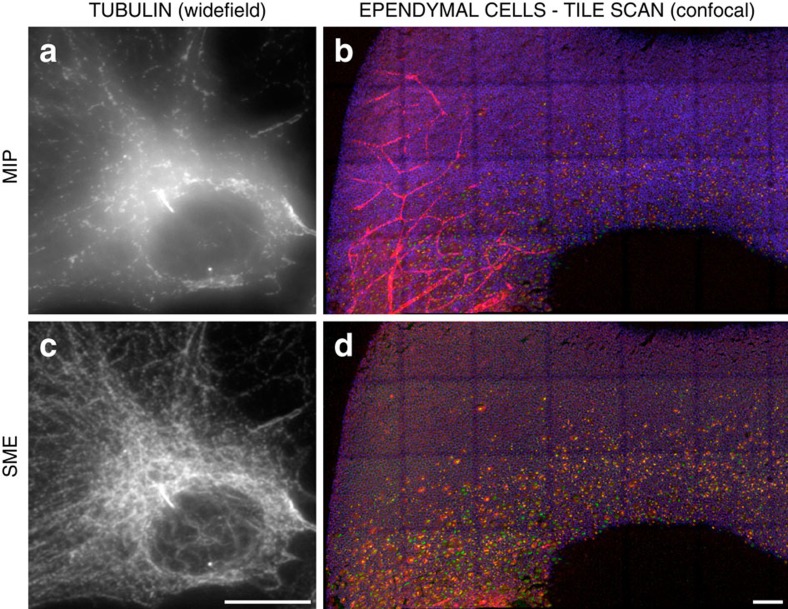
Motivation to preserve spatial consistency within one channel and between channels. (**a**,**c**) An image stack of the immuno-stained tubulin network in a single cell from a study on the formation of tyrosinated tubulin. The stack was imaged by a conventional wide-field microscope and rendered with (**a**) MIP and with (**c**) SME. By selecting a continuous layer of voxels, SME discards the aggregation of irrelevant signal located nearby in the *x*/*y* direction albeit far apart in the *z* direction. A consequence of this selection is an important increase of contrast that enabled distinguishing clearly individual tubulin filaments when compared to the popular MIP approach. Scale bar, 10 μm. (**b**,**d**) Whole-mounted view of a tile scan of ependymal cells from the lateral ventricular surface of a P1 Centrin2gfp transgenic mouse (GFP, green), double immuno-stained with a cell junction marker (βCatenin, blue) and a marker for nascent centriole (*Sas*6, red). For this developmental study of the apical surface, the tissue sample is imaged as multiple stacks stitched together to form a 3D image of 3,929 × 9,307 × 58 voxels. The apical surface being not planar, it was impossible to avoid imaging (in red channel) the blood vessels targeted by the same antibody used for staining nascent centrioles. Consequently, (**b**) MIP, by extracting high-intensity voxels, captures a mix of blood vessels located below the apical surface and of nascent centrioles, which makes any further analysis or even visual inspection difficult. (**d**) SME, by using the cell junction marker channel (blue) as reference, clearly discards the blood vessels that were located below the apical layer, which allow nascent centrioles to be clearly distinguished. Scale bar, 100 μm. A crop of this large slide is presented with more details in Fig. 4d,e. Each data set is further described in Supplementary Table 1.

**Figure 2:**
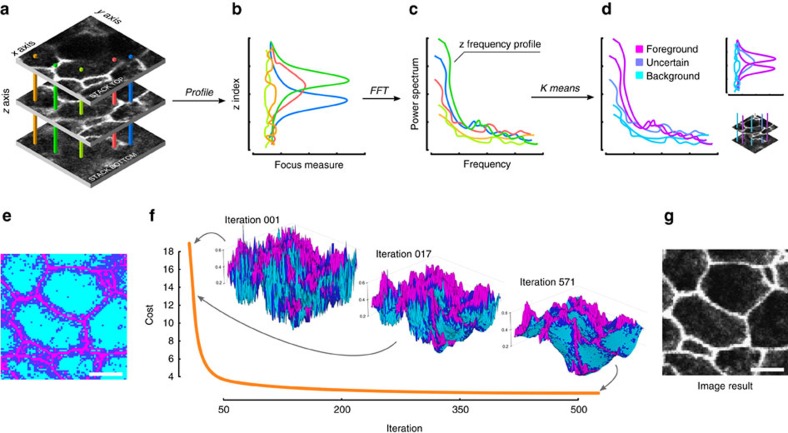
SME algorithm steps. (**a**) Original Image stack. (**b**) Any (*x*,*y*) position corresponds to a profile of focus values in the *z* direction, which is made of direct intensity values in case of confocal image or the SML values for wide-field epifluorescence images. (**c**) Profiles that pass through some foreground signal contain lower-frequency components. Therefore, the power frequency spectrum of each profile is computed using FFT and (**d**,**e**) a three class k-means is performed to associate each (*x*,*y*) position to a label (foreground, background or uncertain). (**f**) A cost function balancing local smoothness and proximity to the maximum of focus value is minimized to obtain the final smoothed index map. Note that the index map, highly discontinuous at the beginning of the optimization process, is smoothed while preserving fine detail on the foreground. (**g**) Finally, voxels corresponding to this index map are extracted from the original stack to produce the final 2D image. Scale bar, 2 μm.

**Figure 3:**
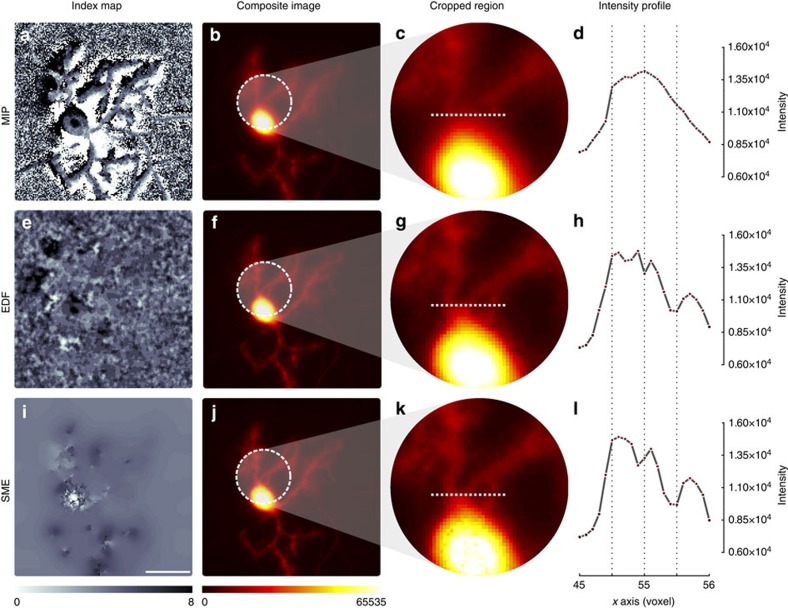
Preserving spatial consistency maintains image resolution. We compare the results obtained by MIP, EDF^9^ and SME on an image stack of a Purkinje cell in 8 days old cerebellar mixed culture acquired by wide-field epifluorescence microscopy to quantify dendritic morphogenesis. The index maps in the first column (**a**,**e**,**i**) show for each pixel (*x*,*y*) the levels in the stack (0–8) from which the corresponding intensity values were extracted to obtain the 2D projection in the second column (**b**,**f**,**j**). Scale bar, 10 μm. The index of MIP shows that two neighbouring pixels can originate from the top and the bottom of the stack and therefore can artificially bring together objects far apart in the 3D volume. EDF, by applying the same local smoothing operator on the index map, reduces this effect but fails to produce a fully continuous index map on the background to preserve foreground details. SME constrains the background to reach the foreground level locally but smooths the foreground much more slightly to preserves details. Zoomed view in column 3 (**c**,**g**,**k**) and intensity profiles in column 4 (**d**,**h**,**l**) show that precision is improved to recover fine details. In this example, the three intensity peaks obtained by SME (**l**) shows a more accurate dendrite quantification by SME, where **d**,**h** could lead to erroneous profiles. This data set named NEURON1 is further described in Supplementary Table 1.

**Figure 4:**
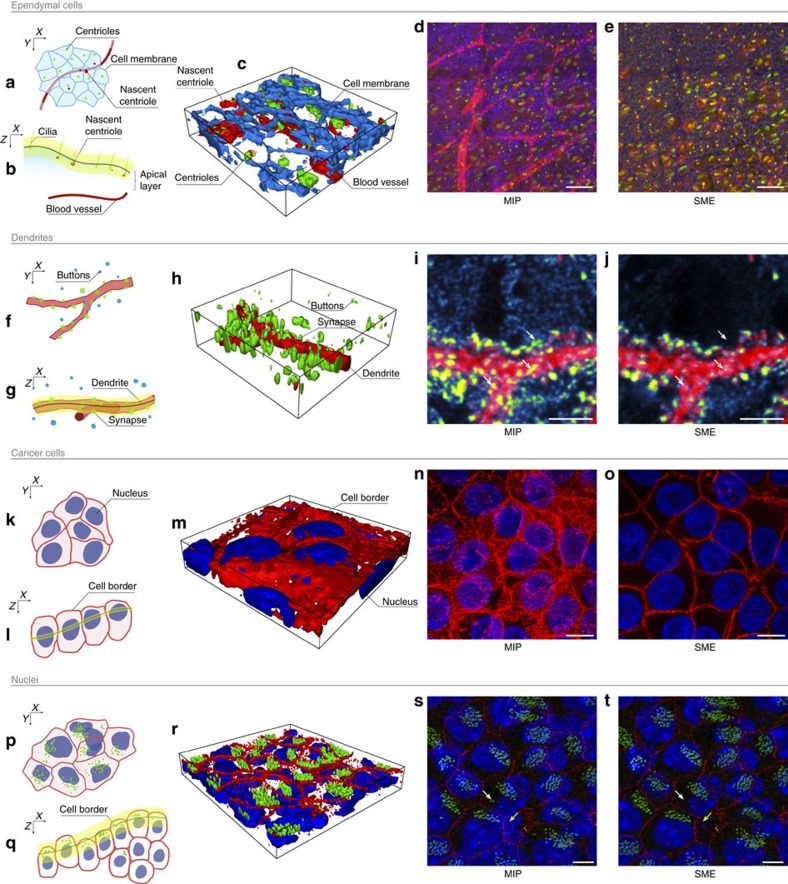
SME improves 2D extraction. (**a**) Apical and (**b**) cross-sectional schematic views of ependymal cells with the extracted manifold in yellow. (**c**) 3D reconstruction where centrioles (green), cell junctions (blue) and nascent centrioles (red) were immuno-stained. (**d**) The MIP fails to distinguish nascent centrioles from the blood vessels located below due to the non-specific *Sas*6 antibody. In contrast, SME (**e**) extracts an image corresponding to the apical surface (cell junction channel given as a reference), making the nascent centrioles visible but avoiding the blood vessels. Scale bar, 50 μm. (**f**,**g**) Schematic views of a dendrite in a cerebellar mixed culture stained for synapse detection. (**h**) 3D reconstruction showing presynaptic boutons from granule cells (green) on Purkinje cell dendritic spines (red). (**i**) MIP projection fails to render the actual co-localization of objects. In contrast, (**j**) by extracting a continuous layer, SME does not bring unrelated boutons artificially close to the dendritic branch as indicated by the white arrows. Scale bar, 5 μm. (**k**,**l**) Schematic views of human breast cancer cells from a study of the co-localization of huntingtin phosphorylation at serine 421 (S421-P-HTT) with E-cadherin cell–cell junction^13^. (**m**) 3D reconstruction of stained nuclei (blue) and cell–cell junction (red). (**n**) MIP prevents to distinguish cell borders. In contrast, (**o**) SME used nuclei stained as reference channel to extract distinguishable cell junctions for further quantification. Scale bar, 10 μm. (**p**,**q**) Schematic views of Cen2GFP adult brain ependymal cells from a study on the beating direction of their cilia. (**r**) 3D reconstruction where nuclei (blue), ZO1 cell–cell junctions (red) and centrioles (green) were stained. (**s**) MIP fuses nuclei located at different depths. In contrast, (**t**) Apical cell–cell junctions stained with ZO1 can be used as reference by SME to extract a manifold passing through the apical layer to allow exclusive identification of individual nuclei located in it (as shown by the white arrow). Scale bar, 5 μm. It also makes the cell junctions clearly visible (as shown by the green arrow). All data sets are further described in Supplementary Table 1.

**Figure 5:**
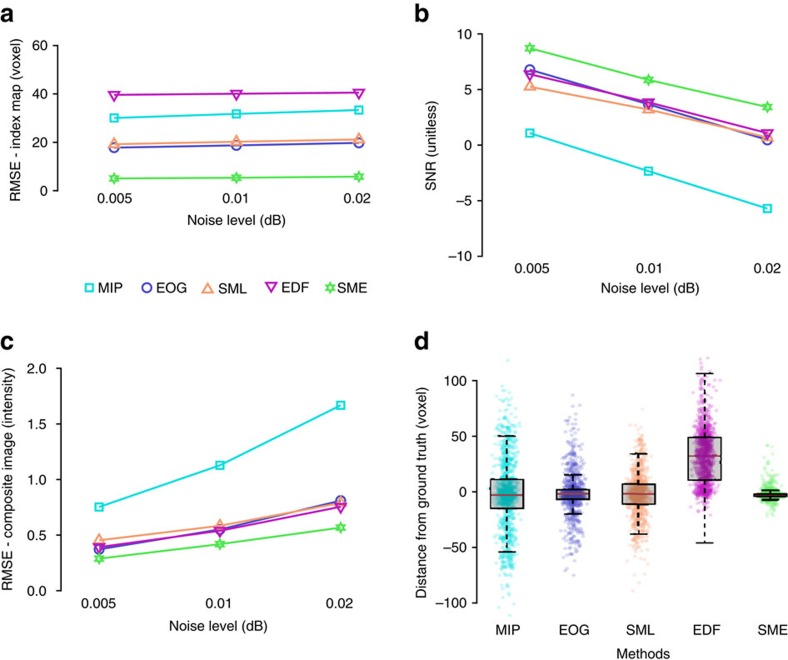
Comparison of SME with four existing methods using synthetic data. We used synthetic image stack (see Supplementary Fig. 7) and compared the results obtained by four different state of the art methods (MIP in light blue, EOG in blue, SML in brown, EDF in purple, see the literature review in the Supplementary Notes for details on each of those methods) and SME (in green). The metrics used for comparison are described in detail in the ‘Methods’ section. (**a**) RMSE on index map quantify the deviation of the extracted manifold from the reference. (**b**) SNR and (**c**) RMSE on composite image measure the quality of the 2D reconstructed image. SME achieves the best combination of high SNR and low RMSE. (**d**) The box plots illustrate the absolute error in the *z* direction (in voxels) between the reference and the reconstructed manifold for a given synthetic data set. A uniformly sub-sampled set of 1,000 pixels is also displayed for each method. We can observe that the departure from the original manifold is significantly smaller in general for SME and extreme values are also further apart for existing methods than for SME.

Fig. 1 describes the motivations of our approach, which is schematized in Fig. 2. In Figs 3 and 4, examples show that existing methods produce artificial spatial relationships between organelles due to the discontinuity of the index map. In contrast, SME clearly discards objects that are not nearby in the original 3D volume by retrieving a continuous layer of voxels. We also compare our approach to accessible and/or state of the art methods by computing objective criteria on synthetic data sets (see Fig. 5 and Supplementary Figs 7 and 8). The results show that SME outperforms existing methods in term of spatial consistency and especially MIP, which is the most used in practice.

### SME preserves image resolution

A consequence of improving spatial consistency is an increased contrast and the unveiling of objects that were not visible using MIP due to the aggregation of the signal from irrelevant layers (see Fig. 1). The advantage is made obvious with epifluorescence wide-field images (see Figs 1a,c and 3) but also while imaging large tissue sample with a confocal microscope as shown by Fig. 1b,d as it would be virtually impossible to obtain a planar acquisition that contains only a single layer of cells along a large epithelium sample. Several other critical examples are presented in Fig. 4 and in Supplementary Figs 9 and 10.

### SME works on wide-field and confocal image stacks

The SME algorithm is similar for both imaging modalities apart from the generation of the initial index map used as a data attachment term in the cost function. Wide-field epi-fluorescence microscopy is fast and efficient to image thin specimen. However, image stacks obtained using that modality always contain significant blur due to the convolution of the emitted fluorescence with the point spread function (PSF) of the microscope, which is wider than for confocal microscopes. Therefore, wide-field image stacks require an adapted focus measure (sum of modified Laplacian) that is based on the second derivatives of the signal rather than on the signal itself (see the results in Figs 1c and 3). While slightly slower in terms of acquisition, confocal microscopes have a drastically reduced PSF blur and therefore are more frequently used to image thicker objects. For that second modality, the focus measure is directly based on the intensity of the signal (see results in Figs 1d and 4 and Supplementary Figs 9 and 10).

### SME preserves consistency between multiple channels

SME applied on a single channel produces a spatially consistent 2D image. To preserve spatial consistency between multiple channels, one of them must be selected as a reference to compute a unique index map, which will be applied to all channels including the reference one. This procedure ensures that a pixel in the resulting 2D image is made of the exact same colour components as the corresponding voxel in the 3D stack preserving consistency between channels. Figure 4 presents several examples where this advantage is emphasized.

### SME is parameter free

The SME cost function parameters are all estimated from the data and the parameters of the optimization process were studied and fixed such as to guarantee convergence for most data sets in a reasonable time (see the ‘Methods’ section and the Supplementary Methods for further details).

## Discussion

Projecting an object from a 3D volume to a 2D plane always results in information loss. The proposed method provides a 2D representation with minimal distortion for objects lying on or close to a 2D manifold embedded in the 3D-observed volume. That is, it assumes the foreground of interest is 2.5D. However, if this hypothesis is not met and the foreground is scattered in the 3D volume or is a full 3D object such as a relatively large spherical cell in suspension, most projection methods would result in severe morphological distortions. While MIP could help getting an overview of the full 3D content, it should be disregarded for any further investigation because, by mixing all layers together, it essentially prevents any reliable geometric interpretation. Similarly, even if the result of SME would not be spatially distorted, it would not be of great interest either as the 2D manifold extracted would represent an incomplete fraction of the surface of the full 3D object or a crossing section of it. Overall, in the case of a full 3D content, a single 2D extraction cannot be satisfactory and an interactive interface that makes possible a 3D exploration should definitely be preferred.

In some applications, one could be interested in ‘looking around’ the actual 2D manifold obtained by SME in the 3D space at the price of distorting slightly the spatial consistency. This is the case for instance when vesicles visualized as spots lies on and around an epithelium. In this case, it can be interesting to collect not only a single layer of voxels corresponding to the smooth index map, but a thicker piece of signal made of more layers around the manifold and aggregated together locally by maximum of intensity. In short, in some case, a local MIP applied in the vicinity of the manifold extracted using SME can be of potential interest. As in practice we ended up using this option for some of our own applications, we made it available in the NIH ImageJ/Fiji plugin.

Selecting a reference channel to compute a unique index map for all channels might be regarded at first as a parameter. However, it is not, it should be a rational choice related to the biological question being tested with a given data set. Indeed, if such a method is used for extraction, it implies that the 3D volume necessarily contains a 2.5D foreground, which in turn implies that the channel of this 2.5D foreground of interest must be specified. For example, in Fig. 1b,d, the surface of interest is the apical layer of ependymal cells, not the incomplete blood vessel network located below. Additional examples illustrating the choice of a rational reference channel are provided in Fig. 4.

In cell biology, the use of 3D stacks has become common practice to image 2.5D objects. We identify that a large proportion of users render stacks by performing a MIP using NIH Fiji/ImageJ. We show that this can lead to wrong interpretation and we propose an easy-to-use solution to obtain a spatially consistent projection within and between image channels. We demonstrate, using synthetic data, that SME outperforms existing methods in term of spatial consistency. Furthermore, we illustrate the impact of this feature on the relevance and the quality of the extraction by applying SME on various biological applications using confocal and wide-field microscopy image stacks. The algorithm is parameter free and is implemented as an NIH ImageJ/Fiji plugin for ease of use. It is also available as open source MATLAB code.

## Methods

### Survey

We conducted an anonymous online survey of microscope users to review common practices with 3D image volumes in the bio-imaging community. The survey shows that a significant fraction of users frequently use 2D projection for the purpose of visualizing 3D data sets and an overwhelming majority (84.3%) of them use the freely available and easy-to-use Fiji Plugin for pixel wise intensity-based Z projection (see Supplementary Figs 1 and 2 for further details).

### Algorithm

The method we propose fit a ‘smooth’, parameter-free, 2D manifold **Z** onto the foreground signal of a chosen reference channel while ‘ignoring’ the background, thus propagating the index map found in the foreground to the local background. This is because, in principle, the foreground is deliberately stained while the background is mostly made of noise and therefore shows random levels of estimated focus. To this aim, the fitting is constrained at each pixel by minimizing together the distance from the map **Z** to the maximum focus map **Z**_max_ (ensuring data attachment) and the local variance of **Z** (ensuring its smoothness). A crucial point in the definition of the cost function to minimize is that, in order for the foreground level of **Z** to propagate to the background, the first term is weighted spatially by the pixel class such that if a pixel belongs to the background this data attachment term is not used, and if the pixel belongs to the foreground or is not clearly defined then a weighting strategy that we described further below is adopted. All together, the method aims at finding the optimal map 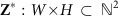
→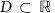 by solving:


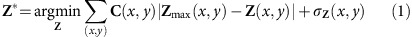


where the maximum focus map **Z**_max_, the weighted class map **C** and the local spatial s.d. *σ*_**Z**_ are defined further in sections below.

### Maximum focus map *Z*_max_

The input image stack is defined as a function that maps positions 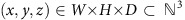 to values 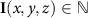. The maximum focus map is then defined as:


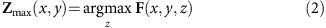


that is, we search the level *z* that maximizes independently at each given (*x*,*y*) co-ordinate the focus measure defined as


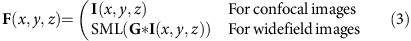


where **I** is the input image and SML stands for sum of modified Laplacian^11^. SML is computed in 2D independently at each level *z* to avoid merging signal from consecutive layers, as resolution in *z* is always worse than in *x*/*y* (ref. 12). Note that original images are convolved by a Gaussian filter **G** prior computing SML. We describe in Supplementary Fig. 3 and in Supplementary Methods the strategy we used to set the s.d. of **G** automatically.

### Weighted class map *C*(*x*,*y*)

A z-profile is defined as the vector of all *z* values at a given (*x*,*y*) location. In the following, the set of all z-profiles of a given volume **Z** is noted **F**_**Z**_(*x*,*y*), where each profile is indexed by its (*x*,*y*) location. In our context where the foreground of interest lies on a 2D manifold embedded in a 3D volume, we identify roughly three types of z-profiles. First, the z-profiles that belong to the foreground contain a relatively low-frequency peak centred on the level of the stained object along with some possible background noise. In the opposite, the z-profiles that belong to the background are rather flat and contain only background noise. Finally, there is an uncertain class that contains profiles that are not clearly defined. Those last profiles are either produced by a dim foreground or they are part of the background but are somehow whitened by the PSF halo of some foreground fluorescence located nearby. To distinguish those three types of profiles independently from their actual peak position, they are converted into power spectra using:


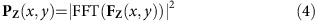


where FFT denotes the fast Fourier transform. A three classes k-means is then performed on those spectral density profiles to roughly identify the three classes: the foreground z-profiles (class with the highest amount of lowest frequency components), the background z-profiles (class with the lowest amount of lowest frequency components) and the uncertain z-profiles (class with intermediate frequency components). This segmentation does not need to be precise as it is not definitive as such but helps driving the final index map towards the foreground. To offer a class-specific control on the relative weight between the regularization term and the data attachment term, a weight **C**(*x*,*y*) is assigned to each z-profile the following way:


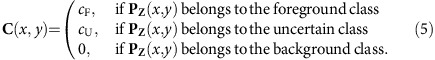


The weight 0 is affected to the background class such that the *z* level for all positions of that class will be determined only by the local curvature and by extension by the local foreground. In the Supplementary Methods and in Supplementary Fig. 4, we show how the weights *c*_F_ and *c*_U_ can be set automatically. Intuitively, if the local curvature of the foreground is large in average and the noise level is low, then *c*_F_ should remain high to preserve the foreground curvature. On the other hand, if the foreground lies on a flattish manifold and the noise level is high, this term should be lower to ensure convergence to a smooth z index map. Hence, an optimal value *c*_F_ exists and depends both on the curvature of the foreground and the noise level. In the cost function (equation 1), decreasing the first term by a given Δ*z* for any location (*x*,*y*) translates to an increase of Δ*σ* and conversely. To scale those two terms on the whole image, we seek for a *c*=Δ*σ*/Δ*z* that would smooth the foreground yet preserving its highest local curvature (the distribution of such *c* on all the voxels of the foreground of an example image is showed in Supplementary Fig. 4b). Taking the maximum value of *c* would prevent any smoothing of the foreground and would be equivalent to consider that there is no noise. On the other hand, taking its average would ensure that the average curvature is maintained, however some regions of high curvature would be overly smoothed, causing partial loss of details. To choose the best compromise, we make the assumption that the noise follows a Bernoulli process, where a pixel of **Z**_max_(*x*,*y*) is either the right *z*-level value on the foreground, or a wrong random value uniformly distributed over [0, *D*] due to the fact a random voxel can be selected as the maximum intensity instead of the correct foreground level (with a higher probability when the signal-to-noise ratio is weak). Fortunately, the probability for the latter to happen can be obtained directly from the misclassification of the maximum intensity values in the foreground class as shown in Supplementary Fig. 4a. Following this idea, *c*_F_ is chosen to be the value of the *c* distribution that match the probability of false positive of the maximum intensity distribution on the foreground profile. This is described in Supplementary Fig. 4c. Once *c*_F_ has been computed, the rational is to choose *c*_U_ between 0 and *c*_F_. Intuitively, if the signal to noise is rather low, then it means that a larger fraction of uncertain profiles in fact belong to the background and should be ignored, *c*_U_ should then be closer to 0. On the opposite, if the signal to noise is rather high, *c*_U_ should be close to *c*_F_. Thus, the correct ratio to apply can be obtained from the relative position of the means of the **Z**_max_ distributions of foreground, background and uncertain classes as showed in Supplementary Fig. 4c.

### Local spatial standard deviation *σ*_**z**_

To enforce the smoothness of the **Z** index map, its local spatial s.d. *σ*_**z**_ computed over a 3 × 3 window around each location (*x*,*y*) is included in the cost function (equation 1) to minimize. Several other local spatial measure of smoothness, as for instance the morphological gradient, could be thought of to play that role but the best results we obtained were achieved using the local spatial s.d.

### Optimization process

The goal of the optimization process is to perform the minimization defined by equation (1) to obtain the final index map Z*. As **Z** is a non-parametric function, a dedicated non-parametric optimization scheme was proposed (see Algorithm 1 in Supplementary Methods). Briefly, starting from an initial index map **Z**^0^=**Z**_max_, the search consists in computing, for each location (*x*,*y*) of the index map **Z**, the cost (equation 1) of moving *z*=**Z**(*x*,*y*) up, down or keeping it steady, and hold the value that minimize it. The algorithm iterates on this simple scheme until a balanced is found between the **Z** map smoothness *σ*_**z**_ and its proximity to the foreground jointly for all pixels. When computing the cost (equation (1)), the maximum focus map **Z**_max_ and the class map **C** are predefined constant. However, the local spatial s.d. *σ*_**z**_ depends on the smoothness of **Z** and needs to be updated whenever **Z** is modified. Therefore, the local spatial s.d. 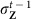 for the whole index map **Z**^*t*−1^ obtained at previous iteration is computed once at the beginning of each iteration on a 3 × 3 widndow. Then, for the sake of computational efficiency, the computation of 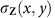 for each location (*x*,*y*) for each shift (up or down) can be computed rapidely from *σ*_**z**_ without the need for rescanning the window (see Supplementary Methods for the details on how the rolling s.d. formula is derived and can be used for this). This minimization process gradually increases the smoothness of the index map while keeping it near the foreground pixels, hence forcing the *z* level of background pixels to shift towards the *z* level of the local foreground. Finally, as the intensity is only known for discrete values of **Z**, at the end of the optimization process the composite image is constructed by extracting the intensity values that are the closest to the **Z** index map on the *z* axis. No interpolation is performed to preserve original fluorescence values. The settings of the two parameters used for this optimization scheme are described in Supplementary Methods and in Supplementary Figs 5 and 6. Also, to intuitively understand the minimization process, Supplementary Movies 1–6 illustrate it visually for six data sets of Supplementary Table 1.

### Evaluation

The performance of the proposed method and the four other approaches mentioned in the literature review (see Supplementary Note 1) were compared quantitatively using synthetic data sets (generated the way described in Supplementary Fig. 7) and real data sets (described in Supplementary Table 1) using the following metrics.

- The RMSE – Index map. The root mean square error between the synthetic index map (**Z**_X_) and the reconstructed index map (**Z**_R_) is computed this way: 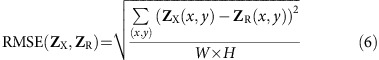 where *W* × *H* is the dimension of the image. This metric quantifies the quality of the manifold reconstruction (see Fig. 5a for results).
- The SNR is the signal-to-noise ratio between the s.d. of the reconstructed intensities *σ*^2^(**I**_R_) and the s.d. of the remaining noise defined as *σ*^2^(**I**_R_−**I**_X_) after reconstruction of the synthetic intensities (**I**_X_). It is computed this way: 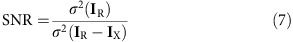 This metric quantifies the ability of a method to remove the noise from the observed image volume (see Fig. 5b for results).
- The RMSE – Composite image. The root mean square error between the synthetic intensities (**I**_X_) and the reconstructed intensity (**I**_R_) is computed this way: 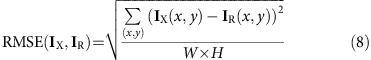 where *W* × *H* is the dimension of the image. This amount quantifies the quality of the recovered composite map (see Fig. 5c for results).
- The Distance from ground truth. It is the distribution of the absolute differences on the *z* axis between the synthetic index map (**Z**_X_) and the reconstructed index map (**Z**_R_). 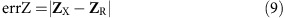 It is also a way to measure the accuracy of the manifold reconstruction (see Fig. 5d for results).

All of these metrics were applied on synthetic data with various level of noise (see Fig. 5). The results show an improvement of SME over the existing methods, whatever the level.

### Software and code

We make the proposed method available as a compiled ImageJ plugin for direct use with ImageJ/Fiji along with the Java code and the equivalent MATLAB scripts at the following address: https://github.com/biocompibens/SME.

### Animals

All animal studies were performed in accordance with the guidelines of the European Community and French Ministry of Agriculture and were approved by the Direction départementale de la protection des populations de Paris (Approval number Ce5/2012/107) and the Comité Régional dEthique en Expérimentation Animale (no. 00057.01) and the veterinary services (C75 05 12).

### Data availability

All 3D image data sets needed to reproduce the results are described in Supplementary Table 1 and available in Harvard Dataverse with the identifier doi:10.7910/DVN/VBFQK8.

## Additional information

**How to cite this article:** Shihavuddin, A. *et al*. Smooth 2D manifold extraction from 3D image stack. *Nat. Commun.* **8**, 15554 doi: 10.1038/ncomms15554 (2017).

**Publisher’s note**: Springer Nature remains neutral with regard to jurisdictional claims in published maps and institutional affiliations.

## Supplementary Material

Supplementary Information Supplementary Figures, Supplementary Table, Supplementary Note, Supplementary Methods and Supplementary References

Supplementary Movie 1 Ependymal Cells. Illustration of the optimization process. The top left panel shows the evolution of the Z index map during the process (or a subset corresponding to the square on the top right panel). The first frame of the animation shows the index map produced by MIP for confocal images or by SML for wide field images (it is used to initialize the process) while the last frame shows the index map obtained by SME (when the minimization is achieved). The top right panel shows the evolution of the composite image corresponding to the Z index map displayed on the left. The bottom left panel is the total cost over all (x,y) positions that can be decomposed into a class-weighted sum of the distance to the MIP Z level (bottom center panel) and the local standard deviation (bottom right panel). All panels are synchronized.

Supplementary Movie 2 Dendrites. Illustration of the optimization process. The top left panel shows the evolution of the Z index map during the process (or a subset corresponding to the square on the top right panel). The first frame of the animation shows the index map produced by MIP for confocal images or by SML for wide field images (it is used to initialize the process) while the last frame shows the index map obtained by SME (when the minimization is achieved). The top right panel shows the evolution of the composite image corresponding to the Z index map displayed on the left. The bottom left panel is the total cost over all (x,y) positions that can be decomposed into a class-weighted sum of the distance to the MIP Z level (bottom center panel) and the local standard deviation (bottom right panel). All panels are synchronized.

Supplementary Movie 3 Membrane 1. Illustration of the optimization process. The top left panel shows the evolution of the Z index map during the process (or a subset corresponding to the square on the top right panel). The first frame of the animation shows the index map produced by MIP for confocal images or by SML for wide field images (it is used to initialize the process) while the last frame shows the index map obtained by SME (when the minimization is achieved). The top right panel shows the evolution of the composite image corresponding to the Z index map displayed on the left. The bottom left panel is the total cost over all (x,y) positions that can be decomposed into a class-weighted sum of the distance to the MIP Z level (bottom center panel) and the local standard deviation (bottom right panel). All panels are synchronized.

Supplementary Movie 4 Neuron 1. Illustration of the optimization process. The top left panel shows the evolution of the Z index map during the process (or a subset corresponding to the square on the top right panel). The first frame of the animation shows the index map produced by MIP for confocal images or by SML for wide field images (it is used to initialize the process) while the last frame shows the index map obtained by SME (when the minimization is achieved). The top right panel shows the evolution of the composite image corresponding to the Z index map displayed on the left. The bottom left panel is the total cost over all (x,y) positions that can be decomposed into a class-weighted sum of the distance to the MIP Z level (bottom center panel) and the local standard deviation (bottom right panel). All panels are synchronized.

Supplementary Movie 5 Neuron 2. Illustration of the optimization process. The top left panel shows the evolution of the Z index map during the process (or a subset corresponding to the square on the top right panel). The first frame of the animation shows the index map produced by MIP for confocal images or by SML for wide field images (it is used to initialize the process) while the last frame shows the index map obtained by SME (when the minimization is achieved). The top right panel shows the evolution of the composite image corresponding to the Z index map displayed on the left. The bottom left panel is the total cost over all (x,y) positions that can be decomposed into a class-weighted sum of the distance to the MIP Z level (bottom center panel) and the local standard deviation (bottom right panel). All panels are synchronized.

Supplementary Movie 6 Tubulin. Illustration of the optimization process. The top left panel shows the evolution of the Z index map during the process (or a subset corresponding to the square on the top right panel). The first frame of the animation shows the index map produced by MIP for confocal images or by SML for wide field images (it is used to initialize the process) while the last frame shows the index map obtained by SME (when the minimization is achieved). The top right panel shows the evolution of the composite image corresponding to the Z index map displayed on the left. The bottom left panel is the total cost over all (x,y) positions that can be decomposed into a class-weighted sum of the distance to the MIP Z level (bottom center panel) and the local standard deviation (bottom right panel). All panels are synchronized.

Supplementary Software The Supplementary Software file is a compressed zip file that contains the Smooth Manifold Extraction plugin for NIH Fiji/ImageJ. Unzip and place the JAR file in the plugins folder of Fiji or ImageJ software to make the "SME Stacking" plugin available from the Fiji/ImageJ Plugins menu. Future versions of the plugin will be available directly from https://github.com/biocompibens/SME.
