# Supplementary material for: Smooth 2D manifold extraction from 3D image stack
Source: Nat Commun. 2017 May 31;8:15554. doi: 10.1038/ncomms15554 (PMC5499208; doi:10.1038/ncomms15554)
Supplement: Supplementary Information — Supplementary Figures, Supplementary Table, Supplementary Note, Supplementary Methods and Supplementary References [file ncomms15554-s1.pdf]

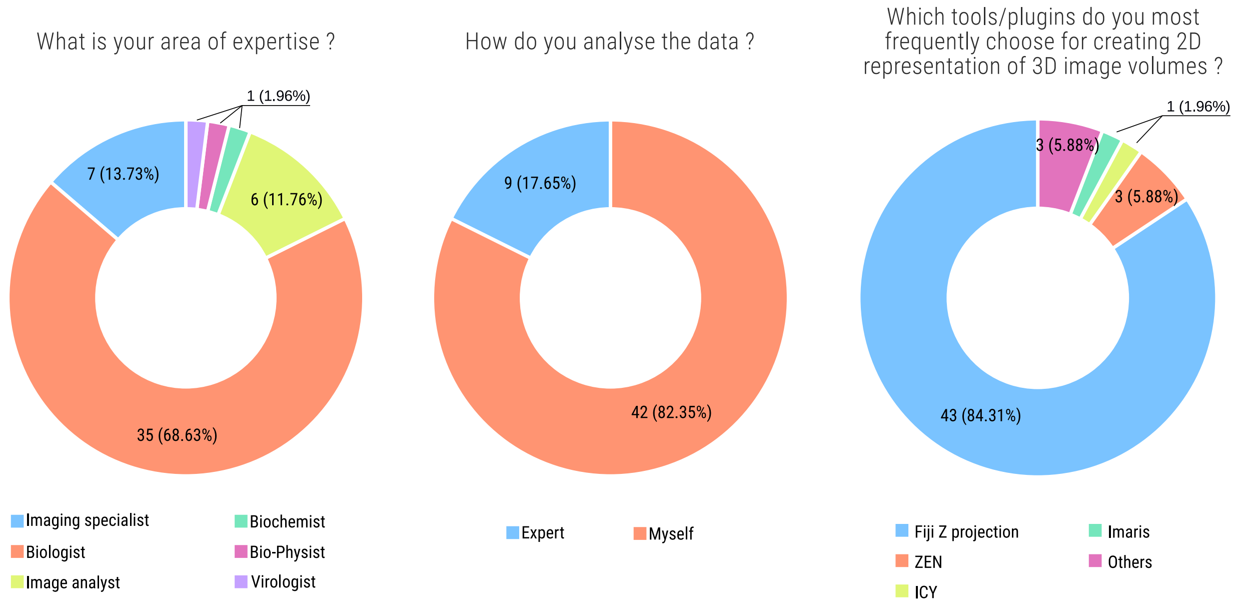

Supplementary Figure 1: **Survey results**. We conducted an anonymous online survey of microscope users to review common practices with 3D image volumes in the bio-imaging community. The questionnaire was created with Google forms and sent to several research teams located at College de France, Ecole Normale Superieure and Pasteur Institute three of the main French research institutions in biology. The questionnaire was mainly propagated among biologists registered with the microscopy platforms of their respective institutions such that to maximize the answer rate from people directly concern with the exploration of 3D image stack. The questions were focused on 3D imaging with the aim of estimating how 3D bio-image data were concretely visualized and analyzed in everyday research in biology. We report here the most relevant findings regarding the scope of the presented work. A significant fraction answered that they frequently use 2D projection for the purpose of visualizing their data and an overwhelming majority (84.3%) of them use the freely available and easy to use Fiji Plugin for pixel wise intensity based Z projection

2D representation of 3D image volume

Specialization

How would you describe your main area of expertise?

- ○ Biologist
- ○ Imaging specialist
- ○ Image analyst
- ○ Other: ______

Do you work with 3D microscopy image stacks?

- ○ yes
- ○ no

For ease of visualization/analysis do you make projections of 3D image volume?

- ○ Always
- ○ Sometimes
- ○ Never

Which tools/plugins do you most frequently choose for creating 2D representation of 3D image volumes?

- ○ Intensity based Z projection (eg Fiji plugin - max of Intensity)
- ○ Derivates of intensity (gradient, edge strengths)
- ○ Wavelets (eg Fiji plugin - Extended depth of field)
- ○ Other: ______

How do you analyse the data?

in others options, names of standard tools expected

- ○ have an expert to do the analysis
- ○ yourself
- ○ Other: ______

How do you analyse the data?

	1	2	3	4	5	
manual	○	○	○	○	○	automatic

Supplementary Figure 2: **Survey form**.

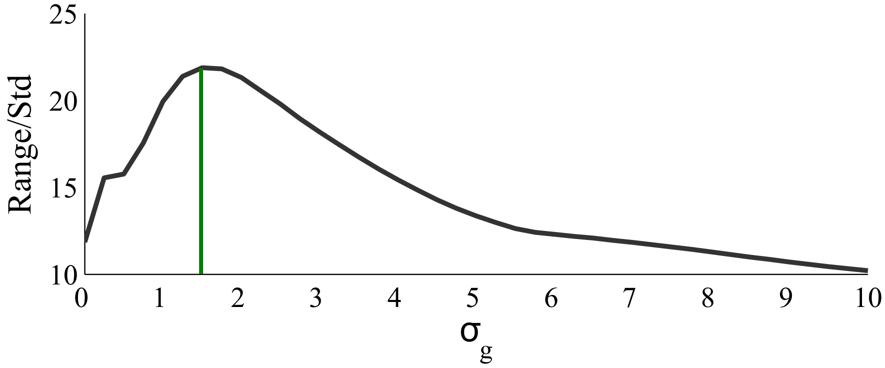

Supplementary Figure 3: **Automated selection of the standard deviation for Gaussian smoothing prior SML computation.** By using SML on stacks acquired with a widefield microscope we aim at enhancing the contrast between the foreground and the background while reducing the noise. Therefore a quantity that makes sense to maximize is the ratio of the difference between the maximum and the minimum value of the SML result image (that we call Range) over the standard deviation of the overall SML result image (that we call Std). By iteratively smoothing a stack obtain by a widefield microscope we can reach a maximum of the given criteria easily. This plot shows a typical evolution of Range/Std obtained by smoothing with a Gaussian kernel with σ from 0 to 10.

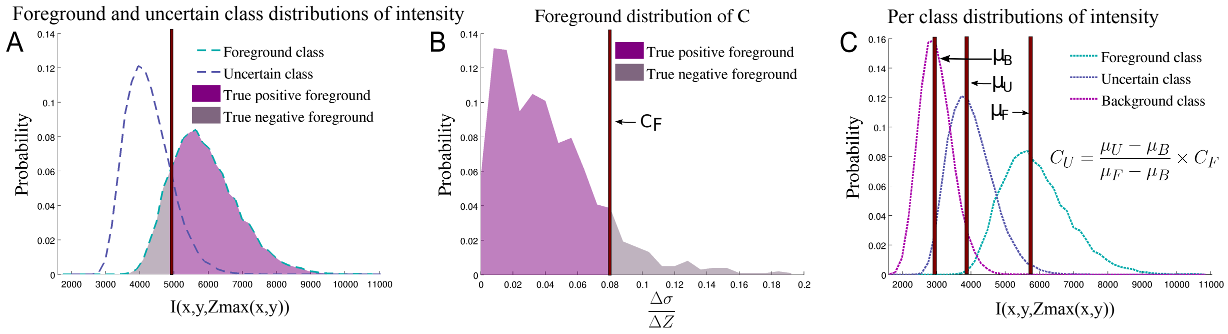

Supplementary Figure 4: **Automated computation of weights c_F and c_U in the weighted class map C.** An optimal c_F minimizing sensitivity to clutter and maximizing sensitivity to the manifold curvature is computed the following way (A) Two $\mathbf{Z}_{\max}$ distributions are computed : one from the foreground class values and one from the uncertain class values defined by the profile classification. An optimal cut-off is automatically obtained at the intersection of both distribution. This cut-off define a balanced ratio of true positive versus true negative foreground. (B) The same ratio is applied to the c distribution computed from the foreground to obtain c_F. (C) The relative position of the means μ_F, μ_U and μ_B of the $\mathbf{Z}_{\max}$ distributions for each class is then used to define a ratio to linearly determine the value of c_U. It is clear here that if μ_U is closer to the background μ_B then the weight for this class will be lower and the minimization will favor the regularization term. In contrast, if μ_U is closer to μ_F the importance of the regularization term will then be lowered.

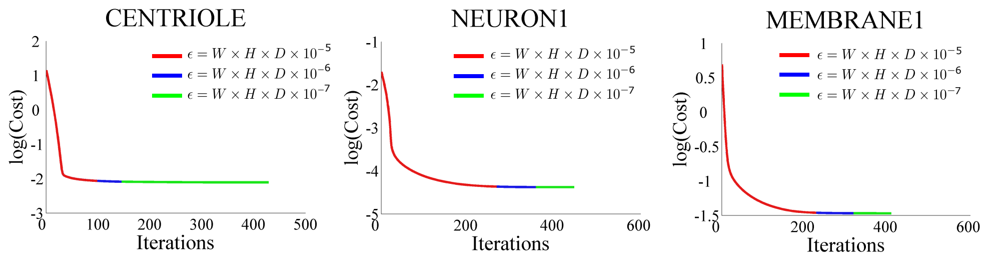

Supplementary Figure 5: **Setting of the stopping criteria** ϵ. The stopping criteria ϵ is set such that additional iterations would not significantly reduce further the cost. The 3 plots presented here show the cost evolution during the minimization process for 3 different choice of ϵ for 3 different datasets. This parameter is robust for all data sets as it is automatically adapted to its size.

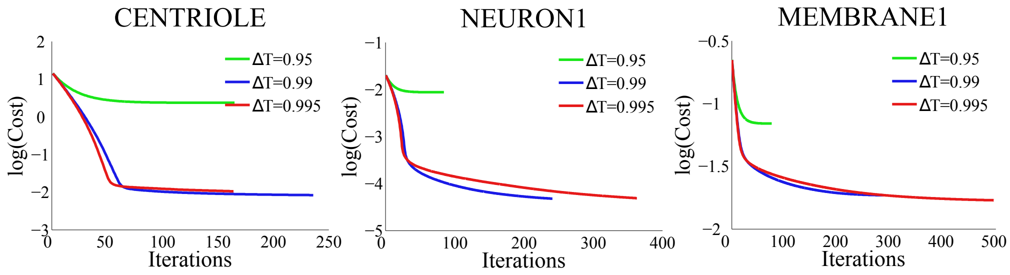

Supplementary Figure 6: **Setting of the decreasing rate** ΔT. We tested various values of ΔT and plot (green, blue and red) the cost minimization in logarithmic scale. We choose the annealing scheme to be guided by $\Delta T = 0.99$ as we observed a slower decrease rate would not lead to a significant additional decrease in cost nor to an improvement in index maps smoothness or image quality. However the value ensured convergence for all the datasets tested.

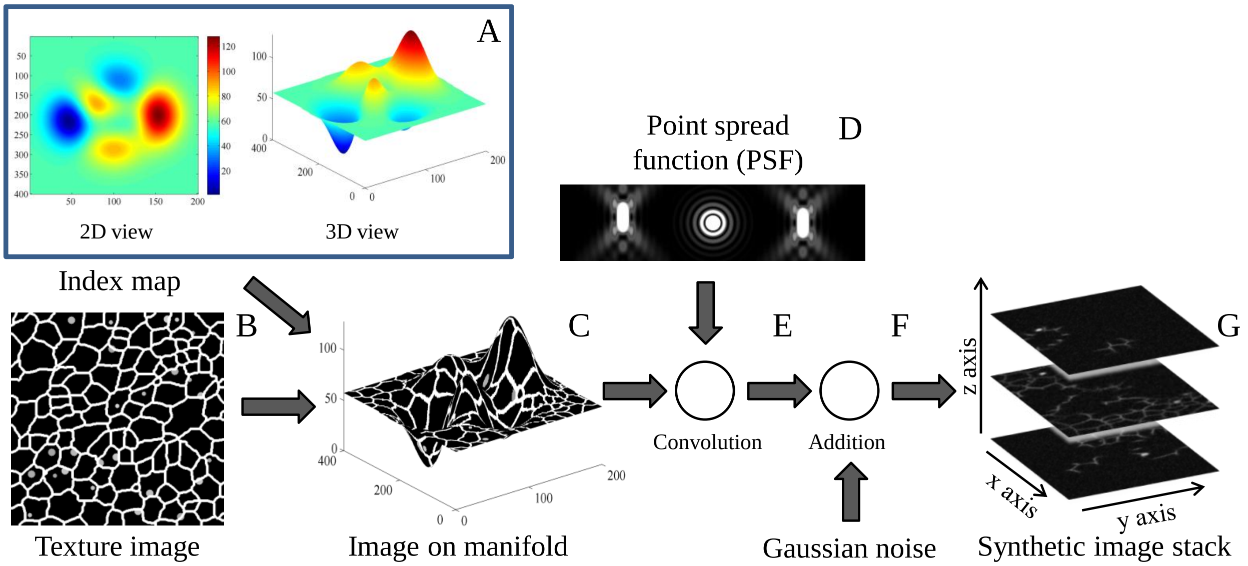

Supplementary Figure 7: **Synthetic data generation**. To perform quantitative comparisons, we created synthetic data that we named SYN-Tissue. To simulate a manifold onto which the objects are lying, (A) a surface made from several polynomials is constructed in a 3D volume (index map) (B) then a reference image (texture image) made of cell borders and randomly located spots to mimic centrioles (C) is fitted on it. (E) Finally the whole 3D image is convolved with (D) the real point spread function (PSF) of a microscope and (F) some noise is added producing the final synthetic image (G) $\mathbf{I}_X$.

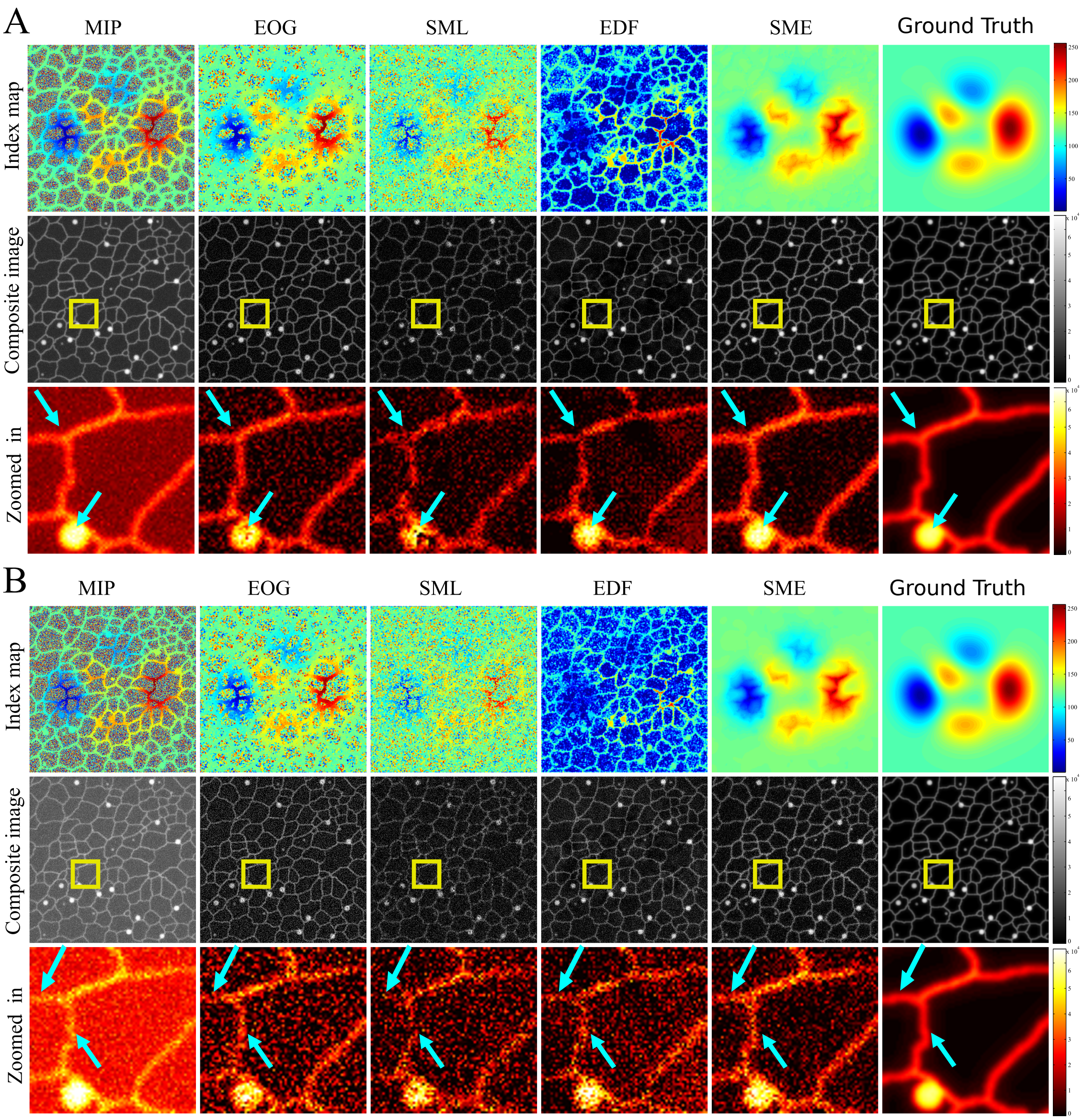

Supplementary Figure 8: **Results on SYN-Tissue image**. Additional Gaussian noise is added with signal to noise (A) 0.001dB and (B) 0.01dB. For each level, top row is the index map represented as a 2D image, middle row is the composite image and bottom row shows zoomed sections highlighting errors and artifacts. The jet colorbar (top row) indicates the stack depth (256 slices), the gray colorbar (middle row) indicates intensity values (16 bits) and the hot colorbar (bottom row) indicates intensity values for the zoomed section (16 bits). From left to right, the 4 methods (MIP, EOG, SML and EDF) - as described in the Literature overview section, are compared to SME. We clearly see in the zoomed results (cyan arrows) that MIP tends to saturate the resulting image causing loss of important details, SML fails to adapt to complex manifold shape and misses essential details. EDF fails to maintain a local structural coherence and the index maps lacks smoothness. Results obtained by SME, the proposed method, are consistently better on those two aspects. Note that the recovered manifold by SME is the closest to the synthetic reference manifold shown in Supplementary Figure 7.

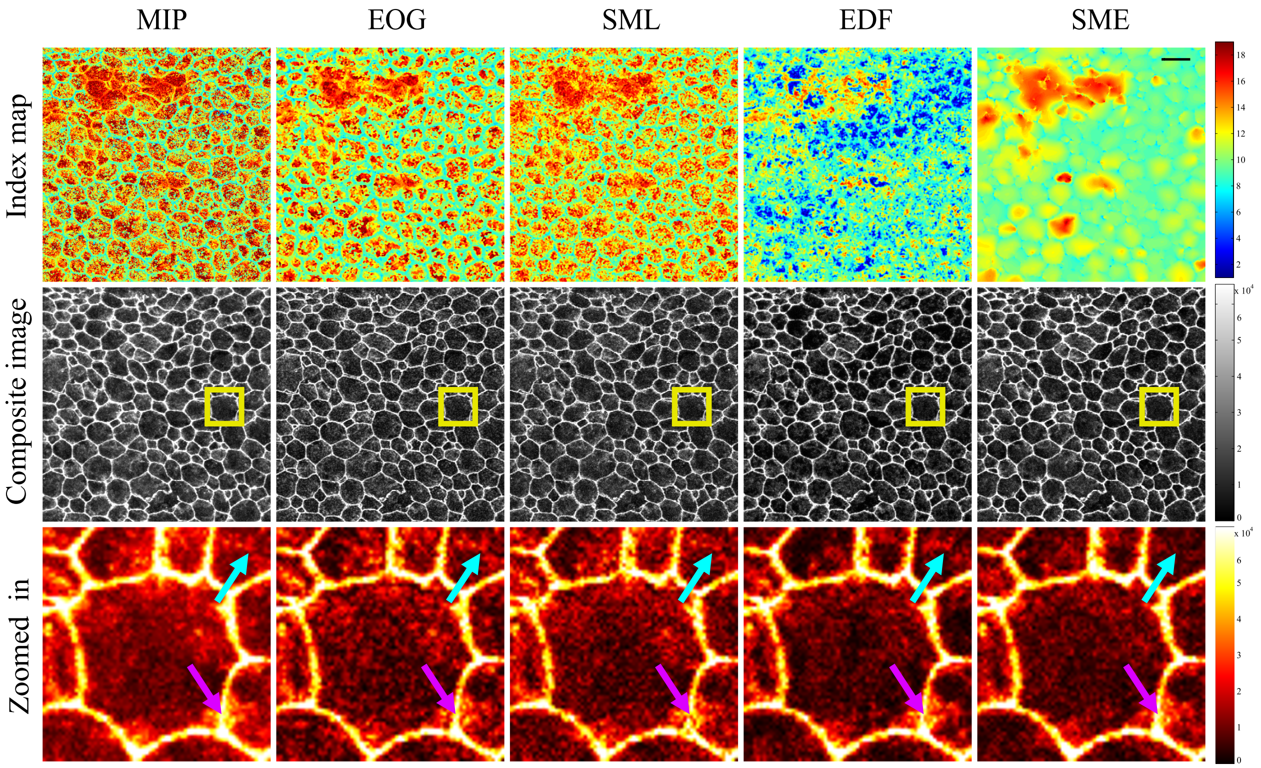

Supplementary Figure 9: **Results on MEMBRANE1**, acquired by confocal microscopy. Facial view of P1 ventricular surface immunostained with cell junctions marker (Z01) Top row is the index map represented as a 2D image, middle row is the composite image and bottom row shows zoomed sections highlighting errors and artifacts. The jet colorbar (top row) indicates the stack depth (19 slices), the gray colorbar (middle row) indicates intensity values (16 bits) and the hot colorbar (bottom row) indicates intensity values for the zoomed section (16 bits). Scale bar is 10 μm. From left to right, the 4 methods (MIP, EOG, SML and EDF) are compared to SME (right most). The top cyan arrow in the zoomed section (bottom row) points to structured noise accumulated by all other methods except SME. The bottom purple arrow shows that cell membranes are render with a higher contrast and a lower amount of noise by the SME method compared to the others. The main reason explaining the higher contrast obtained with SME is that the background voxels selected by SME are not chosen randomly (as in MIP) but they are located close to the foreground, thus the background obtained is less corrupted by the PSF glow that is stronger in the z-direction

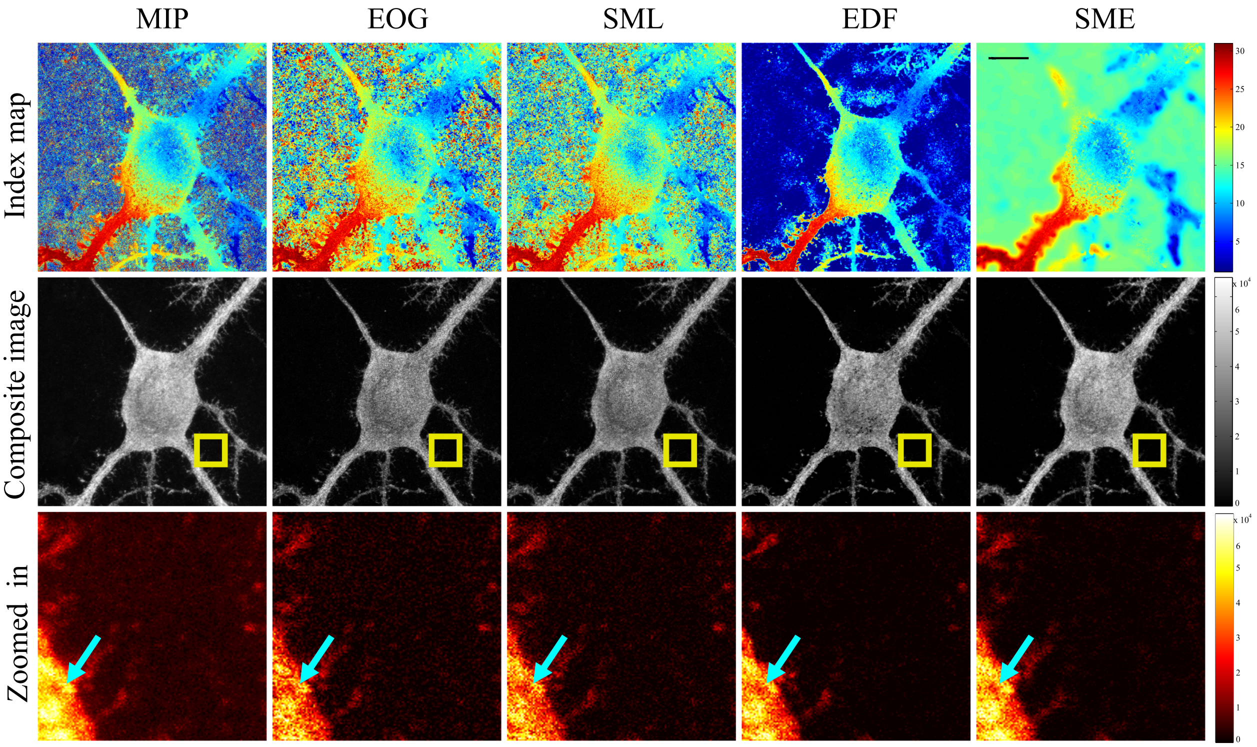

Supplementary Figure 10: **Results on NEURON2 image** acquired by a confocal microscopy. Purkinje cell from a mixed cerebellar culture at day in vitro 7 immunostained using a calbindin antibody and acquired by a confocal microscope. Top row is the index map represented as a 2D image, middle row is the composite image and bottom row shows zoomed sections highlighting errors and artifacts. The jet colorbar (top row) indicates the stack depth (31 slices), the gray colorbar (middle row) indicates intensity values (16 bits) and the hot colorbar (bottom row) indicates intensity values for the zoomed section (16 bits). Scale bar is 10 μm. From left to right, the 4 methods (MIP, EOG, SML and EDF) are compared to SME. Arrows indicate that the other methods tend to create saturated images with possible important loss of texture details. This is also due to the aggregation of voxels originating both from the foreground and the background occasioning possible wrong interpretation.

Supplementary Table 1: **Summary of microscopy data sets**

Thumbnail	Dataset	Subject	Modality	Microscope	Sensor	Objective	Image size	Resolution (in μ^3)	Layer above	Layer below
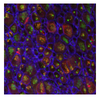	EPENDYMAL CELLS	Postnatal day 1 Centrin2GFP transgenic mouse[1], Ependymal cell junctions stained with βCatenin; nascent centrioles with mouse IgG2b anti-Sas-6 [2]	Confocal	Apotome 2 (Zeiss)	CCD	40x	1024X1024 X58	0.18x0.18 x0.28	15	3
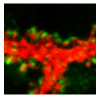	DENDRITES	14 days cerebellar mixed culture, Purkinje cells immunolabeled with calbindin[3], granule cells with VGLUT1	Confocal	Leica SP5	PMT	63x	255X700 X32	0.06x0.06 x0.19	3	3
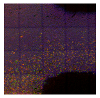	EPENDYMAL CELLS - TILE SCAN	Postnatal day1 Centrin2GFP transgenic mouse[1], Ependymal cell junctions stained with βCatenin; nascent centrioles with mouse IgG2b anti-Sas-6 [2]	Confocal	Leica SP8	Hybrid	40x	3828X5676 X58	0.18x0.18 x0.28	15	3
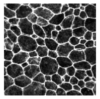	MEMBRANE1	Ependymal cell junctions stained [4] with rabbit anti-ZO1 (Life Technologies)[2]	Confocal	Leica SP8	Hybrid	40x	407X421 X19	0.18x0.18 x0.28	0	0
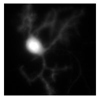	NEURON1	GFP labeled Purkinje cell in 8 days[3] cerebellar mixed culture	Widefield	Leica DMiRBE	CCD	10x	168X201 X8	0.06x0.06 x0.19	0	0
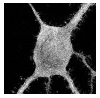	NEURON2	CaPB labeled Purkinje cell in 7 days[3] cerebellar mixed culture	Confocal	Leica SP5	PMT	63x	1024X1024 X31	0.06x0.06 x0.19	0	0
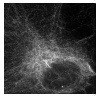	TUBULIN	Primary cycling ependymal progenitor in vitro immunostained for tyrosinated tubulin [2]	Widefield	Apotome 2 (Zeiss)	CCD	100x	556X610 X21	0.065x0.065 x0.23	0	0
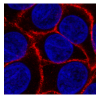	CANCER CELL	Study of colocalization of huntingtin phosphorylation at serine 421 (S421-P-HTT) with cell-cell junction [5] at MCF10 Healthy cells	Confocal	Leica SP5	Hybrid	63x	512X512 X34	0.12x0.12 x0.125	0	0
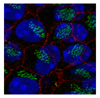	NUCLEI	Centrin2GFP transgenic mouse[1] at P45, Ependymal cell necleus stained with DAPI, junctions stained [4] with rabbit anti-ZO1 (Life Technologies)[2]	Confocal	Leica SP8	Hybrid	63x	370X335 X24	0.121x0.121 x0.21	7	5
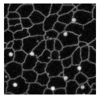	SYNTHETIC TISSUE	Cell junctions and centrioles on a complex synthetic manifold	Confocal	-	-	-	400X400 X128	-	0	0

Supplementary Note 1: literature overview

Microscopy images are rich sources of information for measuring and quantifying biological phenomena. Rapid advances in imaging technology has enabled acquisition of large 3D image volumes. Biological objects that are thicker than the focus range of the microscope lenses are frequently imaged as 3D stacks of 2D images. Optical sectioning of a preparation captures the in-focus information at increasing depths simulating an extended depth of field [8, 6, 9]. A 3D image stack is thus a sequence of focused image slices, which can subsequently be processed to reconstruct a single all-in-focus image. In computational imaging, this technique resulting in an image with greater depth of field (DOF) than any of the individual source images is called focus stacking (alternatively called focal plane merging, z-stacking, focus blending and most commonly z-projection, the most widely adopted methodology for focal stacking). Ideally, a 2D approximation of a 3D volume aims at maximizing information content and object coherence while minimizing the distortion generated by the Point Spread Function (PSF) of the microscope, the noise and the imaging artifacts. Accurate 2D approximation can help subsequent processing and quantification such as segmentation [7] for cell counting or cell profiling [10]. Image deconvolution to restore PSF distortion are commonly used for wide-field images and there are several preconditions on the image acquisition parameters such as the sampling density must be higher than the Nyquist rate of the point spread function (PSF). However, deconvolution is known to create artifacts as if the PSF is not properly modeled, it is then a transformation of the image that is obtained and not a restoration. For deconvolution of 3D image stacks the interested readers may refer to these papers [11, 12]. In the proposed method, we extract a 2D representation based on a smooth 2D manifold embedded in the 3D observed volume on which both the foreground and background are in focus, thus minimizing the PSF effect and other artifacts. In this paper, we employ the term *composite image* for the recovered 2D all-in-focus image and the term *index map* for the 2D image of Z-values indicating the depth at which each pixels information are eventually retrieved from.

In z stacking, various factors such as the chosen focus operator, the depth map smoothing, the window size and the properties of the object imaged (such as the scale variation, the texture etc.) affect the quality of the computed composite image. Focus operators determine which sections of the field of view are in focus at different levels of the stack. In [13, 14, 15], the authors present comprehensive reviews of focus measures. A composite image is obtained by fusion or blending of in-focus pixel values from different slices of an image stack. Spatially coherent smoothing of the depth map has also been extensively studied [16, 17, 18]. In [19], Muhammad and colleagues present a study on the effect of the sampling step size in the z direction on the accuracy of the recovered z-map indices. While the most common methods are Gaussian smoothing and averaging, Bezier surface approximation [20], anisotropic diffusion favoring data guided smoothing [21], nearest neighbor interpolation [22] have also been examined in the context of manifold recovery from focus measure in optical microscopy volumes.

Region based compared to *pixel* based projection prove to be consistently more successful at overcoming artifacts coming from noise and structural incoherence due to mis-aligned or mis-registrated projections [23]. Traditionally, blocks of regular shapes were favored for the ease and simplicity they offered in handling image data. In [24], the authors propose an adaptive window mechanism for image smoothing. In [25], the authors demonstrate how variation in window size affect the accuracy of the depth map estimation and conclude that smaller window sizes lead to a higher accuracy. In [26], the authors reported that an increasing window size reduces the differences in depth map estimation by various focus measures. Overall, it was beneficial to make the projection results free from a given window shape, size and orientation in order to manage regions that are irregularly shaped but semantically significant in the biological context.

In the this section, we examine in detail a few of the existing methods against which we will compare the performance of our proposed method. First, we chose the pixel-wise maximum intensity projection (MIP), the most widely adopted method by biologists due to its availability as a easy to use one-click, parameter free Fiji Plugin. Secondly, we chose a couple of image blending methods with different focus operators as the sum of modified Laplacian (SML) and the energy of gradients (EOG). Among these methods, only MIP is truly pixel based whereas the other methods, EOG, SML and EDF are region based as the focus is obtained from a neighborhood. We observe in our experiments that the critical factor influencing the quality of the

composite image is the index map blending technique chosen to produce a smooth and natural transition in order to preserve spatial coherence. The choice of the focus measure shows a much lower contribution to the overall result is more related to type of microscopy. The last method is the extended depth of field (EDF) also available as a Fiji plugin.

1. **MIP:** Intensity based methods such as maximum intensity projection (MIP) $MIP = \max_z(\mathbf{I})$ are the simplest and most widely used focus criteria (see Supplementary Figure 1). A suite of intensity based Z projection methods are available as Fiji Plugin [27] and seem to be very popular with biologists. The intuition behind is that biologists make a deliberate staining of objects of interest and hence the maxima of intensity represents the location of the object. However, in practise, noise, artifacts and PSF halo often creates intensity peaks in between objects misguiding the index decision map. Intensity based methods, thus, tends to accumulate PSF and noise. Proposed variations of the max are the mean, median, standard deviation or the sum of the pixel values along the slices for each pixel. Although, these create a seemingly less noisy and cleaner composite image, these methods fail to limit artifacts and sharp transitions in index maps.

2. **EOG:** Gradient methods use the first order derivative of image intensities [13]. The intuition driving these methods is that sharp or focused images present stronger gradient compared to blurred, out of focus images. Energy of Gradients (EOG) is computed as $EoG = \max_z(|\nabla(\mathbf{I})|)$ and is the absolute (alternatively squared) magnitude of gradient. Additionally, to induce a spatial coherence, a median or Gaussian smoothing is performed. In our implementation, we chose a Sobel operator for gradient detection followed by a Gaussian smoothing with sigma equal to 1 to obtained the z index map.

3. **SML:** The Sum of Modified Laplacian (SML) as defined in [28] is based on the second derivative of the image intensity. Laplacian based methods were reported to be the most successful in predicting sharpness or focusness of image regions in natural images [13]. Here too, to induce a spatial coherence a Gaussian smoothing with sigma equal to 1 is used to obtained the z index map.

4. **EDF:** In [29], the authors proposed a method which uses wavelet transform co-efficient as a measure of sharpness and then uses a image blending technique to obtain a smooth z index map. It is also available as a Fiji Plugin called the extended depth of field (EDF) and offers the flexibility of choosing among several focus operators (sobel, variance, real wavelets, complex wavelets) and control the amount of smoothing which comes at a trade-off on speed. Although this approach produces better results than the intensity based methods, it fails to maintain smoothness in the transition from background to foreground. Moreover, in case of multi-channel images, it treats channels independently and combine them afterwards. It does not offer the flexibility to treat each channel separately or to chose the channel containing the reference manifold which is most of the time the only relevant option for bio-image analysis.

Supplementary Methods

Parameter settings for the cost function

The cost function we designed contains two parameters (one only in the case of confocal images). In the following we describe how each of those parameters are set automatically from the data to produce a parameter free cost function.

- **Gaussian smoothing prior SML.** A Gaussian smoothing is performed as a preprocessing step of SML for widefield imaging modality. The Supplementary Figure 3 describes how an optimal standard deviation of the Gaussian kernel **G** is obtained directly from the data.

- **Weights c_F and c_U of the class map C.** The purpose of the weighted class map **C** is to balance the two terms of the cost function deferentially depending on the z-profile type. The weight assigned a z-profile that belong to the background is 0 such that no data attachment term is considered in this case. On the other hand, Supplementary Figure 4 described the strategy we designed to obtain optimal values of c_F and c_U, the weights assigned respectively to the z-profiles that belong to the foreground and the background classes.

Parameter settings for the minimization process

The cost function to minimize is now parameter free. However, the minimization process itself requires some fairly common parameters: the termination criteria ϵ and the discretization factor ΔT which the convergence depends on. As we show in this section, the minimization process and the results obtained are robust to the choice of these parameters with some trade-off on speed. Moreover, we adapt those parameters such that they can be set automatically depending on the image size.

- **The termination criteria ϵ.** In Supplementary Figure 5, we evaluated the impact of different ϵ values on several example image datasets (described in Supplementary Table 1). As expected, the experiment shows that the convergence time increase with a smaller ϵ. However the result is not affected as the cost minimization always converge to the same value. In order to maintain a reasonable computing time whatever the data size, ϵ is set to $W \times H \times D \times 10^{-6}$.

- **The discretization factor ΔT.** In Supplementary Figure 6, we evaluated the impact of different ΔT values on several example image datasets (described in Supplementary Table 1). The step size is initialized to $T = D/100$, where D is the number of slices in the image stack. Multiplying it by ΔT at each iteration produces a geometric decay scheme that ensures a faster convergence [30]. Empirically, in all dataset case $\Delta T = 0.99$ gave satisfactory results.

Computation of a rolling standard deviation

If a single value is modified among a set of n values, it is possible to obtain the updated mean (Equation 1) and standard deviation (Equation 2) efficiently from the previous mean and the previous standard deviation respectively. Let's denote $\{X_0, ..., X_{n-1}\}$, the initial set 0, and $\{X_1, ..., X_n\}$ the set 1 obtained from the set 0 by replacing the value X_0 by X_n. The mean μ_1 of Set 1 can be obtained from the mean μ_0 of Set 0 through the difference:

$$\begin{aligned}
\mu_1 - \mu_0 &= \frac{1}{n}\sum_{i=1}^{n} X_i - \frac{1}{n}\sum_{i=0}^{n-1} X_i = \frac{1}{n}\left[\sum_{i=1}^{n-1} X_i + X_n\right] - \frac{1}{n}\left[\sum_{i=1}^{n-1} X_i + X_0\right] = \frac{1}{n}[X_n - X_0] \\
\mu_1 &= \frac{1}{n}[X_n - X_0] + \mu_0 \qquad (1)
\end{aligned}$$

Similarly, the squared standard deviation σ_1^2 of set 1 can be obtained from the squared standard deviation σ_0^2 of the set 0 by using the following definition of the sample variance:

$$\begin{aligned}
\sigma_0^2 &= \frac{1}{n}\sum_{i=0}^{n-1} X_i^2 - \mu_0^2 \\
\sigma_1^2 &= \frac{1}{n}\sum_{i=1}^{n} X_i^2 - \mu_1^2
\end{aligned}$$

and writing the difference

$$\begin{aligned}
\sigma_1^2 - \sigma_0^2 &= \frac{1}{n}\left[\sum_{i=1}^{n} X_i^2 - \sum_{i=0}^{n-1} X_i^2\right] + (\mu_0^2 - \mu_1^2) \\
\sigma_1^2 - \sigma_0^2 &= \frac{1}{n}\left[\sum_{i=1}^{n-1} X_i^2 + X_1^2 - \sum_{i=1}^{n-1} X_i^2 - X_0^2\right] + (\mu_0^2 - \mu_1^2) \\
\sigma_1^2 &= \frac{1}{n}\left[X_1^2 - X_0^2\right] + (\mu_0^2 - \mu_1^2) + \sigma_0^2 \qquad (2)
\end{aligned}$$

This rolling standard deviation formula (Equation 2) is used in the algorithm to test each shift in $\mathbf{Z}(x, y)$ (in brackets below the argmin in Algorithm 1) for each position (x, y) inside the loop, without having to rescan the whole window around (x, y) to compute the standard deviation.

Algorithm 1 SME: Smooth Manifold Extraction

Require: 3D Image stack $\mathbf{I}$ with dimension $W \times H \times D$,
Ensure: Projected 2D Image with dimension $W \times H$

Compute the maximum focus map $\mathbf{Z}_{\max}$.
Compute the power frequency profiles $\mathbf{P_Z}$.
Perform a 3 class *k-means* of the power frequency profiles $\mathbf{P_Z}$.
Construct class map $\mathbf{C}$ and $\mathbf{Z}_{\max}$ distributions per class.
Initialize $t = 1$, $\mathbf{Z}^0 = \mathbf{Z}_{\max}$, $T_0 = D/100$, $\epsilon = W \times H \times D \times 10^{-6}$ and $\Delta T = 0.99$
do
  $\mu_{\mathbf{Z}}^{t-1} \leftarrow$ Compute local spatial mean of $\mathbf{Z}^{t-1}$ with a window size $n = 3 \times 3$
  $\sigma_{\mathbf{Z}}^{t-1} \leftarrow$ Compute local spatial standard deviation of $\mathbf{Z}^{t-1}$ with a window size $n = 3 \times 3$
  for all $(x, y) \in W \times H$ **do**
    $\mathbf{Z}^t(x,y) \leftarrow \underset{\mathbf{Z}(x,y) \in \left\{ \begin{array}{l} \mathbf{Z}^{t-1}(x,y)-T_t, \\ \mathbf{Z}^{t-1}(x,y), \\ \mathbf{Z}^{t-1}(x,y)+T_t \end{array} \right\}}{\operatorname{argmin}} \mathbf{C}(x,y) \left|\mathbf{Z}_{\max}(x,y) - \mathbf{Z}(x,y)\right| + \sigma_{\mathbf{Z}}(x,y)$
    with $\sigma_{\mathbf{Z}}(x,y) = \sqrt{\frac{1}{n}\left[\mathbf{Z}(x,y)^2 - \mathbf{Z}^{t-1}(x,y)^2\right] + \left[\mu_{\mathbf{Z}}^{t-1}(x,y)^2 - \mu_{\mathbf{Z}}(x,y)^2\right] + \sigma_{\mathbf{Z}}^{t-1}(x,y)^2}$
    and $\mu_{\mathbf{Z}}(x,y) = \frac{1}{n}\left[\mathbf{Z}(x,y) - \mathbf{Z}^{t-1}(x,y)\right] + \mu_{\mathbf{Z}}^{t-1}(x,y)$
  end for
  $T_t \leftarrow T_t \times \Delta T$
  $t \leftarrow t + 1$
while $|E(\mathbf{Z}^t) - E(\mathbf{Z}^{t-1})| > \epsilon$
Return projected image $\mathbf{I}(x, y, \mathbf{Z}^t(x, y))$

Supplementary References

[1] Higginbotham, H., Bielas, S., Tanaka, T. & Gleeson, J. G. Transgenic mouse line with green-fluorescent protein-labeled Centrin 2 allows visualization of the centrosome in living cells. *Transgenic Res.* **13**, 155–164 (2004).

[2] Al Jord, A. *et al.* Centriole amplification by mother and daughter centrioles differs in multiciliated cells. *Nature* **516**, 104–107 (2014).

[3] Sigoillot, S. M. *et al.* The Secreted Protein C1QL1 and Its Receptor BAI3 Control the Synaptic Connectivity of Excitatory Inputs Converging on Cerebellar Purkinje Cells. *Cell reports* **10(5)**, 820–832(2015).

[4] Foerster, P. *et al.* mtorc1 signaling and primary cilia are required for brain ventricle morphogenesis. *Development* dev–138271 (2016).

[5] Thion, M. S. *et al.* Unraveling the role of huntingtin in breast cancer metastasis. *Journal of the National Cancer Institute* **107**, djv208 (2015).

[6] Aguet, F., Van De Ville, D. & Unser, M. Model-based 2.5-d deconvolution for extended depth of field in brightfield microscopy. *Image Processing, IEEE Transactions on* **17**, 1144–1153 (2008).

[7] Tsanov, N., *et al.* smiFISH and FISH-quant–a flexible single RNA detection approach with super-resolution capability. *Nucleic Acids Research* **44(22)**, 165 (2016).

[8] Dowski, E.R. Cathey, W.T. Extended depth of field through wave-front coding. *Applied optics.* **34(11)**, 1859–1866 (1995).

[9] Thériault, G. *et al.* Extended two-photon microscopy in live samples with Bessel beams: steadier focus, faster volume scans, and simpler stereoscopic imaging. *Frontiers in cellular neuroscience.* **8**, 139 (2014).

[10] Ljosa, V. *et al.* Comparison of methods for image-based profiling of cellular morphological responses to small-molecule treatment. *J. Biomol. Screen.* **18**, 1321–1329 (2013).

[11] Sibarita, J.-B. Deconvolution microscopy. In *Microscopy Techniques*, 201–243 (Springer, 2005).

[12] Sage, D. *et al.* Deconvolutionlab2: An open-source software for deconvolution microscopy. *Methods* (2017).

[13] Pertuz, S., Puig, D. & Garcia, M. A. Analysis of focus measure operators for shape-from-focus. *Pattern Recognition* **46**, 1415–1432 (2013).

[14] Huang, W. & Jing, Z. Evaluation of focus measures in multi-focus image fusion. *Pattern Recognition Letters* **28**, 493–500 (2007).

[15] Smith, M. I. & Heather, J. P. A review of image fusion technology in 2005. In *Defense and Security*, 29–45 (International Society for Optics and Photonics, 2005).

[16] Malik, A. S. & Choi, T.-S. Analysis of effects of texture reflectance and source illumination on focus measures for microscopic images. In *Computer and Electrical Engineering, 2009. ICCEE'09. Second International Conference on*, vol. 2, 529–532 (IEEE, 2009).

[17] Marturi, N., Dembélé, S. & Piat, N. Depth and shape estimation from focus in scanning electron microscope for micromanipulation. In *International Conference on Control, Automation, Robotics & Embedded Systems, CARE'13.*, 1–6 (2013).

[18] Shim, S.-O., Malik, A. S. & Choi, T.-S. Accurate shape from focus based on focus adjustment in optical microscopy. *Microscopy research and technique* **72**, 362–370 (2009).

[19] Muhammad, M. S. & Choi, T.-S. Sampling for shape from focus in optical microscopy. *Pattern Analysis and Machine Intelligence, IEEE Transactions on* **34**, 564–573 (2012).

[20] Muhammad, M. S. & Choi, T.-S. A novel method for shape from focus in microscopy using bezier surface approximation. *Microscopy research and technique* **73**, 140–151 (2010).

[21] Boshtayeva, M., Hafner, D. & Weickert, J. A focus fusion framework with anisotropic depth map smoothing. *Pattern Recognition* **48(11)**, 3310–3323 (2015).

[22] Zhao, H., Shang, Z., Tang, Y. Y. & Fang, B. Multi-focus image fusion based on the neighbor distance. *Pattern recognition* **46**, 1002–1011 (2013).

[23] Piella, G. A general framework for multiresolution image fusion: from pixels to regions. *Information fusion* **4**, 259–280 (2003).

[24] Goshtasby, A. & Satter, M. An adaptive window mechanism for image smoothing. *Computer Vision and Image Understanding* **111**, 155–169 (2008).

[25] Malik, A. S. & Choi, T.-S. Consideration of illumination effects and optimization of window size for accurate calculation of depth map for 3d shape recovery. *Pattern Recognition* **40**, 154–170 (2007).

[26] Mahmood, M. T., Majid, A. & Choi, T.-S. Optimal depth estimation by combining focus measures using genetic programming. *Information Sciences* **181**, 1249–1263 (2011).

[27] Schindelin, J. *et al.* Fiji: an open-source platform for biological-image analysis. *Nature methods* **9**, 676–682 (2012).

[28] Nayar, S. K. & Nakagawa, Y. Shape from focus. *IEEE Trans. Pattern Anal. Mach. Intell.* **16**, 824–831 (1994).

[29] Forster, B., Van De Ville, D., Berent, J., Sage, D. & Unser, M. Complex wavelets for extended depth-of-field: A new method for the fusion of multichannel microscopy images. *Microscopy Research and Technique* **65**, 33–42 (2004).

[30] Kirkpatrick Jr, S., Delatt, C.D., Vecchi, M.P. Optimization by simulated annealing.. *Science* **220(4598)**, 671–680 (1983).
